# Temporal trends in acute care costs of hip fracture treatment from 2011 to 2021 in Japan

**DOI:** 10.1007/s11657-025-01607-3

**Published:** 2025-09-12

**Authors:** Masaki Hatano, Hideo Yasunaga, Hisatoshi Ishikura, Takeyuki Tanaka, Shotaro Aso, Sakae Tanaka

**Affiliations:** 1 Department of Clinical Epidemiology and Health Economics, School of Public Health, The University of Tokyo, 7-3-1 Hongo, Bunkyo ku, Tokyo 113-0033 Japan; 2 Department of Orthopaedic Surgery, Faculty of Medicine, The University of Tokyo, 7-3-1 Hongo, Bunkyo-Ku, Tokyo 113-0033 Japan; 3 Department of Health Services Research, Graduate School of Medicine, The University of Tokyo, 7-3-1 Hongo, Bunkyo-Ku, Tokyo 113-0033 Japan

**Keywords:** Hip fracture, Length of stay, Economic burden, Cost

## Abstract

**Summary:**

We investigated annual trends in acute care costs for hip fracture treatment in Japan from 2011 to 2021. While gross medical costs and costs per hospitalization initially declined, both increased after 2013 and 2014, respectively. In contrast, daily medical costs per person rose steadily throughout the study period.

**Purpose:**

Hip fractures impose a substantial financial burden on healthcare systems, primarily due to acute-care hospitalization costs. Despite efforts over the past decade to improve hospital efficiency, trends in acute care costs for hip fracture treatment remain unclear. This study aimed to evaluate annual trends in these costs in Japan.

**Methods:**

We conducted a nationwide retrospective cohort study using the Diagnosis Procedure Combination database in Japan. Patients aged ≥ 60 years who underwent hip fracture surgery between 2011 and 2021 were included. Primary outcomes were gross medical costs, costs per hospitalization, and daily medical costs per person. Secondary outcomes were length of hospital stay and waiting times for surgery.

**Results:**

A total of 839,179 hip fracture cases were identified. Gross medical costs decreased from $890 million in 2011 to $830 million in 2013 but increased to $989 million in 2021. Medical costs per hospitalization declined from $11,587 in 2011 to $11,337 in 2014 and rose to $12,019 in 2021. In contrast, daily medical costs per person increased steadily from $378 to $442 over the study period. Both the mean hospital stay (37.1 to 33.1 days) and waiting times for surgery (3.5 to 3.0 days) decreased from 2011 to 2021.

**Conclusion:**

Although hospital efficiency improved, initial decreases in gross costs and costs per hospitalization were followed by gradual increases after 2013 and 2014, respectively. Meanwhile, daily medical costs per person consistently increased, underscoring the sustained economic burden of acute hip fracture care.

**Supplementary Information:**

The online version contains supplementary material available at 10.1007/s11657-025-01607-3.

## Introduction

Hip fractures represent a substantial economic burden on healthcare systems worldwide. Japan is at the forefront of global demographic shifts driven by population aging. The number of elderly patients in Japan has increased [1], and consequently, the demand for healthcare resources is expected to rise [2, 3]. The acute management of hip fractures typically involves surgery and hospitalization, both of which are major contributors to inpatient costs. Notably, Japan’s healthcare system is characterized by prolonged hospital stays, extended waiting times for surgery, and greater reliance on inpatient rehabilitation than other countries [2]. These factors further exacerbate resource constraints in an aging population. In response, several initiatives have been introduced over the past decade to improve hospital efficiency, including efforts to reduce hospital stays, shorten waiting times for surgery, and enhance the regional distribution of medical services [3, 4]. Concurrently, optimal management strategies for hip fractures have evolved to emphasize multidisciplinary care, timely surgical intervention, and structured rehabilitation programs aimed at restoring mobility and preventing future fractures. Internationally, the Fragility Fracture Network, a global learning organization, has issued a Call to Action to improve care for individuals with fragility fractures by promoting coordinated, evidence-based clinical systems [5]. In Japan, the establishment of the Fragility Fracture Network–Japan in 2015 has similarly facilitated the dissemination and implementation of such strategies nationwide [6]. Another notable initiative in Japan is the"Toyama Model,"an integrated care pathway that ensures seamless transitions from acute care to rehabilitation and community-based support. This model has achieved both improved patient outcomes and cost containment [7]. Collectively, these international and domestic efforts have the potential to enhance recovery, reduce complications, and alleviate the overall healthcare burden associated with hip fractures.

Nonetheless, the impact of these initiatives on inpatient costs for hip fracture treatment over time remains unclear. While improvements in hospital efficiency could help curb costs, other factors—such as population aging, advancements in treatment techniques, and changing patterns of healthcare resource utilization—may also influence cost trajectories [8, 9]. Additionally, cost variations linked to patient demographics and hospital characteristics have yet to be thoroughly examined. A comprehensive understanding of these trends is critical for informing healthcare resource allocation and ensuring the financial sustainability of hip fracture management. Moreover, insights from this study may serve as valuable guidance for other countries confronting similar demographic pressures.

## Purpose

This study aimed to examine annual trends in inpatient costs for hip fracture treatment in Japan from 2011 to 2021. Additionally, we sought to explore how these trends vary based on patient and institutional factors to better elucidate the cost dynamics of acute hip fracture care.

## Materials and methods

### Study design and settings

This retrospective cohort study utilized the Diagnosis Procedure Combination (DPC) database, a nationally representative inpatient database in Japan. Details of the database have been described previously [10]. Briefly, it contains discharge abstracts and administrative claims data from more than 1,000 acute-care hospitals, covering over 50% of all acute-care inpatients. Participation was mandatory for 82 academic hospitals, while community hospitals joined voluntarily [10]. The database includes patient-level data such as age, sex, smoking status, body mass index (BMI), level of consciousness at admission, and pre-admission settings, including private homes, other hospitals, and nursing or care facilities. It also records main diagnoses, admission-precipitating diagnoses, comorbidities present on admission, and complications arising during hospitalization, coded using the International Classification of Diseases, 10th Revision (ICD-10) and recorded as text in Japanese. Additionally, the database contains detailed records of daily and surgical procedures based on Japanese procedure codes and total hospitalization costs. A prior validation study demonstrated high specificity and sensitivity for procedure data and high specificity with moderate sensitivity for diagnoses within this database [11]. This study received approval from the appropriate Research Ethics Committee [approval number: 3501-(5), May 19th, 2021]. The requirement for informed consent was waived due to the anonymized nature of the database.

### Study patients

We identified adults aged ≥ 60 years with either a main diagnosis or an admission-precipitating diagnosis of hip fracture (ICD-10 codes S72.0, S72.1) who underwent surgery—hemiarthroplasty, total hip arthroplasty, or open reduction internal fixation—between April 1, 2011, and March 31, 2022. The original Japanese procedure codes used for inclusion criteria are listed in Supplemental Table 1. Patients were excluded if they had diagnoses of multiple traumas, open fractures, or pathological fractures at admission, or if surgery occurred more than 14 days after admission. ICD-10 codes for the exclusion criteria are provided in Supplemental Table 2.

### Outcomes and covariates

Primary outcomes included trends in gross medical costs, medical costs per hospitalization, daily medical costs, and a breakdown of medical costs per hospitalization. Secondary outcomes were the length of hospital stay, waiting times for surgery, activities of daily living (ADL) at discharge, in-hospital mortality, discharge patterns, the implementation of musculoskeletal rehabilitation during hospitalization, and the initiation of musculoskeletal rehabilitation within one day after surgery. Medical costs were calculated using reference prices from the fee schedule, which specifies itemized charges for hospitalization, consultations, oral drugs, injections, laboratory tests, radiological examinations, surgery and anesthesia, and other procedures performed during hospitalization. Gross medical costs represent the total aggregated expenditure for all eligible cases in each calendar year. Medical cost per hospitalization refers to the total reimbursement claimed for a single acute care admission related to hip fracture. Daily medical cost denotes the average cost per inpatient day, calculated by dividing the total hospitalization cost by the length of stay for each case.

Hospitalization fees were determined based on a combination of DPC code-specific per-diem points, length of hospital stay, and facility-specific coefficients. Costs associated with meals during hospitalization were excluded. Since the number of hospitals enrolled in the DPC system varied over the years, annual gross medical costs and the number of hip fracture cases were adjusted based on patient registration numbers in the DPC system [12]. Additionally, medical costs were adjusted for inflation using Japan’s 2020 consumer price index to enable year-to-year comparisons [13]. These adjusted costs were then converted to US dollars using the exchange rate on February 2, 2025 (155.19 yen per 1 USD).

We compared 2019 international data on the mean length of hospital stay and waiting times for surgery [14–16]. ADL at discharge was assessed using the Barthel Index score. Discharge patterns were categorized as discharge to home, discharge to care facilities, or transfer to another hospital, including rehabilitation hospitals.

Patient demographic and clinical characteristics included age, sex, BMI, impaired consciousness at admission, smoking status (current smoker), source of admission, fracture type, type of surgery, each component of the Charlson Comorbidity Index, anesthetic technique (general, spinal, or general plus epidural anesthesia), and whether surgery was performed on a weekend. BMI was categorized as < 18.5, 18.5–24.9, 25.0–29.9, and ≥ 30.0 kg/m^2^. Impaired consciousness was defined as a Japan Coma Scale score of ≥ 1 on admission. The Japan Coma Scale (JCS) is used to assess the level of consciousness. A score of JCS 0 indicates a fully alert state; scores of 1 to 3 (1-digit) denote patients who are awake without external stimulation; scores of 10 to 30 (2-digit) represent patients who can be aroused by some stimuli; and scores of 100 to 300 (3-digit) indicate coma. Source of admission was classified as home, care facilities, or transfer from another hospital. Admission source data were available starting in 2014.

### Statistical analysis

Continuous variables are expressed as means with standard deviations, and categorical variables as numbers and percentages. Descriptive statistics were calculated for demographic and clinical characteristics and study outcomes. Temporal trends were analyzed using the Cochran–Armitage trend test for proportions and the Jonckheere–Terpstra trend test for continuous or ordinal variables.

Subgroup analyses were performed to evaluate medical costs per hospitalization and daily medical costs, stratified by institution type (academic vs. non-academic hospitals), sex, and age groups (60–69, 70–79, 80–89, and ≥ 90 years). Academic hospital status was defined by whether the institution served as a primary university hospital. Additionally, a post hoc analysis examined the distribution of medical cost breakdowns by institutional level. All statistical analyses were conducted using Stata version 18 software (StataCorp, College Station, Texas, United States).

## Results

A total of 839,179 hip fracture cases were identified between 2011 and 2021. Patient characteristics are summarized in Table 1. The adjusted number of hip fractures decreased from 76,836 cases in 2011 to 71,429 in 2013, before increasing to 82,272 in 2021 (p < 0.001). The mean patient age rose from 82.9 years (SD 8.1) in 2011 to 84.3 years (SD 8.3) in 2021 (p < 0.001). The proportion of male patients increased from 20.4% in 2011 to 22.8% in 2021 (p < 0.001). Similarly, the proportion of patients presenting with impaired consciousness at admission increased from 11.7% to 20.4% (p < 0.001). Admissions from care facilities also rose, from 18.0% in 2014 to 20.0% in 2021 (p < 0.001). In contrast, the percentage of patients with a Charlson Comorbidity Index of 0 declined from 64.3% in 2011 to 54.8% in 2021 (p < 0.001). The proportion of patients undergoing arthroplasty (including hemiarthroplasty and total hip arthroplasty) and those receiving general anesthesia increased over the study period, from 34.2% and 45.9% in 2011 to 39.7% and 61.5% in 2021, respectively (p < 0.001 for both comparisons).

**Table 1:** Trends in patient characteristics for hip fracture, 2011–2021

	2011	2012	2013	2014	2015	2016	2017	2018	2019	2020	2021	p-value
Number of hip fractures	N = 67,658	N = 67,286	N = 65,967	N = 77,356	N = 79,061	N = 84,739	N = 82,221	N = 81,263	N = 76,298	N = 80,789	N = 76,541	< 0.001
Adjusted number of hip fractures	N = 76,836	N = 72,464	N = 71,429	N = 73,795	N = 74,086	N = 76,372	N = 76,583	N = 75,994	N = 75,649	N = 84,109	N = 82,272	< 0.001
Age, mean (SD)	82.9 (8.1)	82.9 (8.2)	83.1 (8.2)	83.2 (8.2)	83.5 (8.2)	83.6 (8.2)	83.7 (8.2)	83.9 (8.2)	84.0 (8.3)	84.2 (8.3)	84.3 (8.3)	< 0.001
Sex (male), n (%)	13,799 (20.4%)	13,941 (20.7%)	13,600 (20.6%)	16,351 (21.1%)	16,847 (21.3%)	18,064 (21.3%)	18,156 (22.1%)	18,012 (22.2%)	16,965 (22.2%)	18,218 (22.6%)	17,440 (22.8%)	< 0.001
**Body mass index, n (%)**												< 0.001
< 18.5	18,043 (29.8%)	17,912 (29.3%)	17,559 (29.1%)	20,882 (29.3%)	21,557 (29.4%)	22,976 (29.2%)	22,134 (28.9%)	21,744 (28.6%)	20,125 (28.3%)	21,892 (29.0%)	20,485 (28.7%)	
18.5–24.9	36,284 (60.0%)	37,004 (60.4%)	36,415 (60.4%)	42,810 (60.1%)	44,108 (60.1%)	47,110 (59.9%)	45,969 (60.1%)	45,792 (60.3%)	42,966 (60.4%)	45,228 (59.9%)	42,903 (60.1%)	
25.0–29.9	5,442 (9.0%)	5,592 (9.1%)	5,561 (9.2%)	6,723 (9.4%)	6,843 (9.3%)	7,468 (9.5%)	7,424 (9.7%)	7,449 (9.8%)	7,062 (9.9%)	7,382 (9.8%)	7,045 (9.9%)	
≥ 30.0	680 (1.1%)	707 (1.2%)	776 (1.3%)	856 (1.2%)	929 (1.3%)	1,039 (1.3%)	992 (1.3%)	999 (1.3%)	958 (1.3%)	1,046 (1.4%)	977 (1.4%)	
Impaired consciousness at admission	7,885 (11.7%)	8,399 (12.5%)	8,896 (13.5%)	11,562 (14.9%)	12,593 (15.9%)	13,506 (15.9%)	13,555 (16.5%)	14,402 (17.7%)	14,557 (19.1%)	16,487 (20.4%)	15,794 (20.6%)	< 0.001
Smoking status (current smoker), n (%)	12,654 (18.7%)	12,993 (19.3%)	12,820 (19.4%)	14,768 (19.1%)	14,571 (18.4%)	15,799 (18.6%)	15,906 (19.3%)	15,697 (19.3%)	15,542 (20.4%)	16,689 (20.7%)	16,587 (21.7%)	< 0.001
**Source of admission**												< 0.001
Home				56,804 (73.4%)	57,437 (72.6%)	61,470 (72.5%)	60,126 (73.1%)	58,940 (72.5%)	54,943 (72.0%)	58,604 (72.5%)	55,369 (72.3%)	
Another hospital				6,136 (7.9%)	6,247 (7.9%)	6,621 (7.8%)	6,321 (7.7%)	6,465 (8.0%)	6,160 (8.1%)	5,928 (7.3%)	5,613 (7.3%)	
Care facilities				13,890 (18.0%)	14,929 (18.9%)	16,183 (19.1%)	15,463 (18.8%)	15,656 (19.3%)	15,068 (19.7%)	16,134 (20.0%)	15,454 (20.2%)	
Others				526 (0.7%)	448 (0.6%)	465 (0.5%)	311 (0.4%)	202 (0.2%)	127 (0.2%)	123 (0.2%)	105 (0.1%)	
**Charlson Comorbidity Index, n (%)**												< 0.001
0	43,491 (64.3%)	42,709 (63.5%)	41,033 (62.2%)	46,767 (60.5%)	47,779 (60.4%)	49,129 (58.0%)	45,620 (55.5%)	44,669 (55.0%)	42,235 (55.4%)	44,128 (54.6%)	41,955 (54.8%)	
1	5,363 (7.9%)	5,296 (7.9%)	5,222 (7.9%)	5,941 (7.7%)	5,993 (7.6%)	6,111 (7.2%)	5,911 (7.2%)	5,744 (7.1%)	5,325 (7.0%)	5,609 (6.9%)	5,461 (7.1%)	
2	14,698 (21.7%)	15,021 (22.3%)	15,457 (23.4%)	19,189 (24.8%)	19,679 (24.9%)	21,986 (25.9%)	23,118 (28.1%)	22,991 (28.3%)	21,487 (28.2%)	23,006 (28.5%)	21,954 (28.7%)	
3	1,809 (2.7%)	1,872 (2.8%)	1,790 (2.7%)	2,298 (3.0%)	2,419 (3.1%)	2,972 (3.5%)	2,907 (3.5%)	3,071 (3.8%)	2,885 (3.8%)	3,204 (4.0%)	2,934 (3.8%)	
≥ 4	2,297 (3.3%)	2,388 (3.5%)	2,465 (3.6%)	3,161 (3.9%)	3,191 (3.9%)	4,541 (5.5%)	4,665 (5.6%)	4,788 (5.9%)	4,366 (5.6%)	4,842 (6.1%)	4,237 (5.5%)	
**Fracture type, n (%)**												< 0.001
Femoral neck fracture	36,639 (54.2%)	35,698 (53.1%)	34,703 (52.6%)	40,379 (52.2%)	40,961 (51.8%)	43,445 (51.3%)	41,983 (51.1%)	41,512 (51.1%)	39,264 (51.5%)	41,427 (51.3%)	39,827 (52.0%)	
Femoral trochanteric fracture	31,019 (45.8%)	31,588 (46.9%)	31,264 (47.4%)	36,977 (47.8%)	38,100 (48.2%)	41,294 (48.7%)	40,238 (48.9%)	39,751 (48.9%)	37,034 (48.5%)	39,362 (48.7%)	36,714 (48.0%)	
**Type of surgery, n (%)**												< 0.001
Hemiarthroplasty	22,727 (33.6%)	22,747 (33.8%)	22,442 (34.0%)	26,640 (34.4%)	27,344 (34.6%)	29,420 (34.7%)	28,924 (35.2%)	28,772 (35.4%)	27,162 (35.6%)	29,508 (36.5%)	28,800 (37.6%)	
Total hip arthroplasty	377 (0.6%)	414 (0.6%)	458 (0.7%)	623 (0.8%)	721 (0.9%)	957 (1.1%)	1,074 (1.3%)	1,385 (1.7%)	1,526 (2.0%)	1,566 (1.9%)	1,591 (2.1%)	
Open reduction internal fixation	44,554 (65.9%)	44,125 (65.6%)	43,067 (65.3%)	50,093 (64.8%)	50,996 (64.5%)	54,362 (64.2%)	52,223 (63.5%)	51,106 (62.9%)	47,610 (62.4%)	49,715 (61.5%)	46,150 (60.3%)	
Cemented arthroplasty, n (%)	3,579 (5.3%)	3,256 (4.8%)	2,884 (4.4%)	3,389 (4.4%)	3,311 (4.2%)	3,469 (4.1%)	3,590 (4.4%)	3,644 (4.5%)	3,493 (4.6%)	3,993 (4.9%)	4,500 (5.9%)	
**Anesthesia, n (%)**												< 0.001
General anesthesia	31,073 (45.9%)	32,526 (48.3%)	33,204 (50.3%)	40,571 (52.4%)	42,007 (53.1%)	46,477 (54.8%)	46,203 (56.2%)	47,553 (58.5%)	45,363 (59.5%)	49,378 (61.1%)	47,106 (61.5%)	
Spinal anesthesia	31,205 (46.1%)	29,877 (44.4%)	28,700 (43.5%)	32,131 (41.5%)	32,442 (41.0%)	33,786 (39.9%)	31,890 (38.8%)	30,381 (37.4%)	28,148 (36.9%)	28,571 (35.4%)	26,900 (35.1%)	
General anesthesia + Epidural anesthesia	2,534 (3.7%)	2,298 (3.4%)	1,967 (3.0%)	2,147 (2.8%)	2,076 (2.6%)	2,030 (2.4%)	1,859 (2.3%)	1,731 (2.1%)	1,365 (1.8%)	1,392 (1.7%)	1,182 (1.5%)	
Others	2,846 (4.2%)	2,585 (3.8%)	2,096 (3.2%)	2,507 (3.2%)	2,536 (3.2%)	2,446 (2.9%)	2,269 (2.8%)	1,598 (2.0%)	1,422 (1.9%)	1,448 (1.8%)	1,353 (1.8%)	
Weekend day surgery, n (%)	2,939 (4.3%)	2,977 (4.4%)	2,841 (4.3%)	3,345 (4.3%)	3,514 (4.4%)	3,601 (4.2%)	3,529 (4.3%)	2,960 (3.6%)	3,208 (4.2%)	3,138 (3.9%)	2,965 (3.9%)	< 0.001

### Primary outcomes

**Fig. 1:**
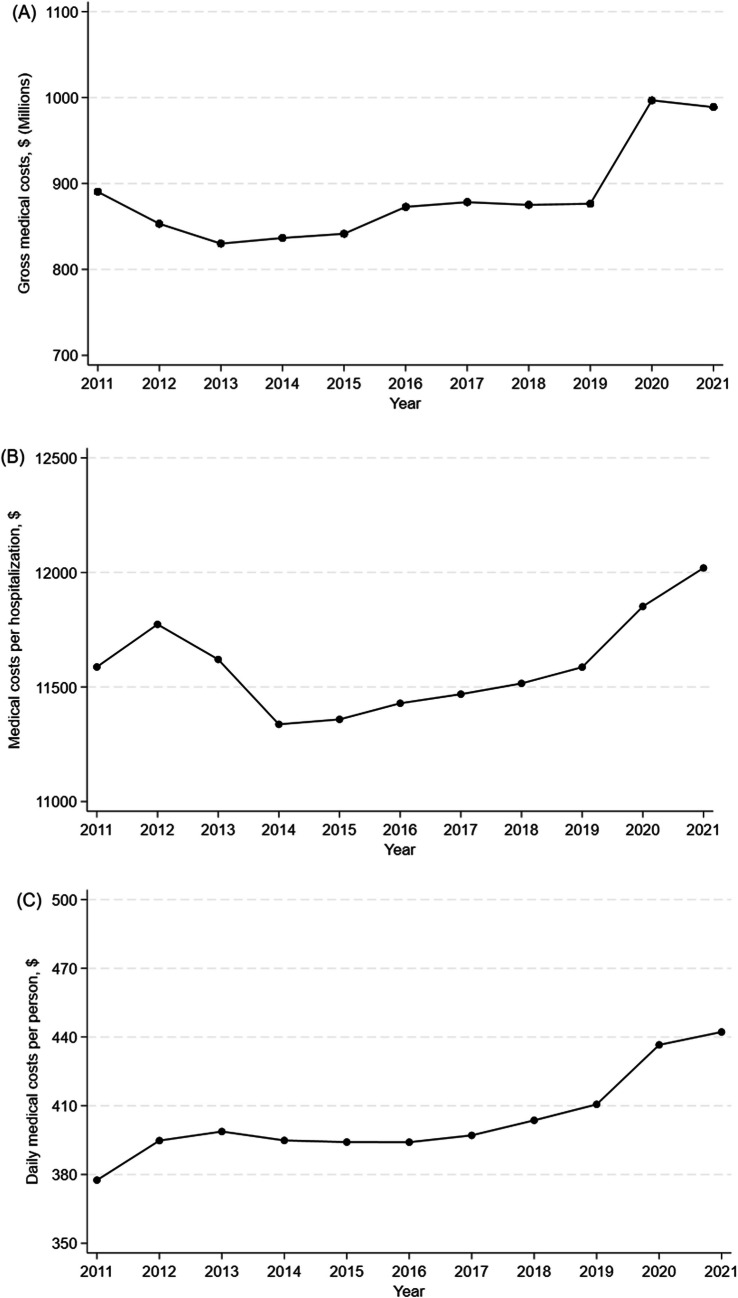
Annual changes in medical costs, 2011–2021.**a** Annual changes in gross medical costs. **b** Annual changes in medical costs per hospitalization. **c** Annual changes in daily medical costs per person

Gross medical costs for hip fracture treatment decreased from $890 million in 2011 to $830 million in 2013 but subsequently increased to $989 million in 2021 (p < 0.001) (Fig. 1A). Medical costs per hospitalization declined from $11,587 in 2011 to $11,337 in 2014, then rose to $12,019 in 2021 (p < 0.001) (Fig. 1B). Daily medical costs per person steadily increased from $378 in 2011 to $442 in 2021 (p < 0.001) (Fig. 1C). During the study period, costs associated with oral medications and injections decreased slightly (p < 0.001 for both), while costs for laboratory tests, radiological examinations, and other resources increased (p < 0.001 for all comparisons). Trends in hospitalization fees closely paralleled those of medical costs per hospitalization (p < 0.001) (Table 2).

**Table 2:** Trends in secondary outcomes, 2011–2021

	2011	2012	2013	2014	2015	2016	2017	2018	2019	2020	2021	p-value
**Mean (SD) of medical cost breakdown per hospitalization, US$**												
Consultation fee	143 (111)	138 (113)	138 (109)	134 (109)	136 (106)	131 (114)	130 (106)	132 (106)	133 (109)	138 (106)	143 (112)	0.414
Oral drugs	62 (126)	64 (123)	66 (205)	61 (129)	60 (163)	55 (143)	55 (131)	53 (206)	52 (143)	51 (181)	51 (146)	< 0.001
Injection	195 (566)	185 (621)	184 (654)	166 (618)	163 (483)	154 (1053)	139 (486)	125 (454)	123 (383)	130 (1343)	128 (826)	< 0.001
Procedure	136 (648)	142 (811)	142 (2295)	124 (587)	123 (630)	119 (586)	117 (794)	115 (631)	112 (563)	112 (677)	113 (644)	< 0.001
Surgery and anesthesia	4662 (2239)	4794 (2163)	4742 (2198)	4586 (2047)	4459 (1970)	4353 (1846)	4364 (1923)	4319 (1834)	4349 (1885)	4427 (1928)	4478 (1947)	< 0.001
Laboratory tests	314 (230)	326 (241)	326 (240)	322 (216)	328 (228)	333 (222)	338 (222)	338 (218)	339 (218)	384 (241)	441 (253)	< 0.001
Radiological examination	214 (169)	225 (172)	230 (171)	230 (275)	237 (164)	244 (158)	247 (152)	249 (149)	252 (143)	267 (148)	269 (145)	< 0.001
Other resources	718 (996)	751 (1080)	748 (1172)	753 (1101)	772 (1141)	820 (1229)	822 (1256)	860 (1489)	838 (1284)	879 (1305)	864 (1281)	< 0.001
Hospital fee	5156 (3153)	5161 (3137)	5055 (3167)	4971 (2971)	5090 (3350)	5228 (3386)	5265 (3582)	5335 (3419)	5398 (3486)	5473 (3643)	5541 (3593)	< 0.001
**Discharge pattern, n (%)**												< 0.001
Home	26,355 (39.0%)	24,690 (36.7%)	22,939 (34.8%)	22,399 (29.0%)	22,387 (28.3%)	24,450 (28.9%)	23,331 (28.4%)	22,876 (28.2%)	20,642 (27.1%)	22,188 (27.5%)	21,020 (27.5%)	
Other hospitals	31,446 (46.5%)	32,828 (48.8%)	33,067 (50.1%)	39,569 (51.2%)	40,382 (51.1%)	43,080 (50.8%)	42,249 (51.4%)	41,946 (51.6%)	40,096 (52.6%)	41,989 (52.0%)	39,346 (51.4%)	
Care facilities	7,027 (10.4%)	7,612 (11.3%)	7,801 (11.8%)	13,950 (18.0%)	14,931 (18.9%)	15,700 (18.5%)	15,192 (18.5%)	14,978 (18.4%)	14,184 (18.6%)	15,195 (18.8%)	14,683 (19.2%)	
Others	2,830 (4.2%)	2,156 (3.2%)	2,160 (3.3%)	1,438 (1.9%)	1,361 (1.7%)	1,509 (1.8%)	1,449 (1.8%)	1,463 (1.8%)	1,376 (1.8%)	1,417 (1.8%)	1,492 (1.9%)	
In-hospital mortality, n (%)	1,231 (1.8%)	1,173 (1.7%)	1,174 (1.8%)	1,277 (1.7%)	1,234 (1.6%)	1,431 (1.7%)	1,372 (1.7%)	1,389 (1.7%)	1,337 (1.8%)	1,371 (1.7%)	1,454 (1.9%)	0.354
Mean (SD) of Barthel index score	55.8 (31.8)	54.7 (31.7)	54.1 (31.8)	53.5 (31.9)	53.5 (31.9)	53.6 (31.9)	53.8 (32.1)	53.5 (32.0)	53.5 (31.9)	52.4 (31.9)	52.7 (32.1)	< 0.001
Musculoskeletal rehabilitation, n (%)	66,297 (98.0%)	65,978 (98.1%)	65,098 (98.7%)	76,575 (99.0%)	78,321 (99.1%)	83,958 (99.1%)	81,440 (99.1%)	80,479 (99.0%)	75,594 (99.1%)	79,950 (99.0%)	75,748 (99.0%)	< 0.001
Musculoskeletal rehabilitation initiated within 1 day after surgery, n (%)	40,853 (60.4%)	42,543 (63.2%)	43,666 (66.2%)	53,588 (69.3%)	56,558 (71.5%)	62,068 (73.2%)	61,186 (74.4%)	63,229 (77.8%)	59,838 (78.4%)	64,772 (80.2%)	62,217 (81.3%)	< 0.001

### Secondary outcomes

**Fig. 2:**
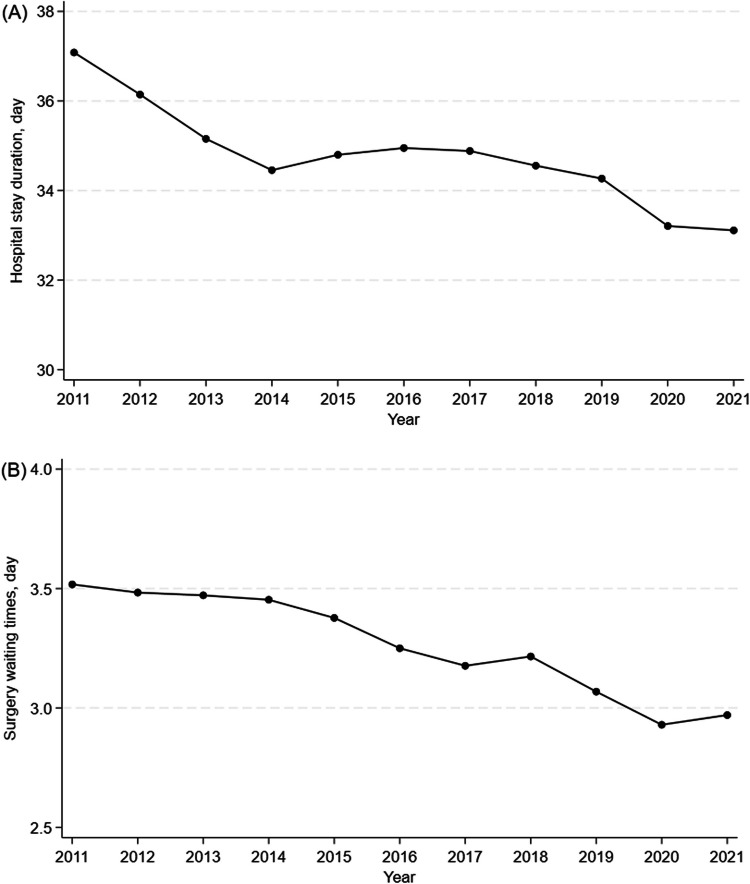
Annual changes in length of hospital stay and waiting times for surgery, 2011–2021. **a** Annual changes in length of hospital stay. **b** Annual changes in waiting times for surgery

Between 2011 and 2021, the mean hospital stay decreased from 37.1 to 33.1 days, and the mean surgical waiting times shortened from 3.5 to 3.0 days (p < 0.001 for both) (Figs. 2A and 2B). In-hospital mortality remained relatively unchanged during this period (p = 0.354) (Table 2). The Barthel Index score at discharge declined slightly, from 55.8 in 2011 to 52.7 in 2021 (p < 0.001) (Table 2). Discharge patterns shifted over time: the proportion of patients discharged to home decreased from 39.0% in 2011 to 27.5% in 2021, while discharges to care facilities, transfers to other hospitals increased from 10.4% and 46.5% in 2011 to 19.2% and 51.4% in 2021, respectively (p < 0.001 for all) (Table 2). Moreover, both the implementation of musculoskeletal rehabilitation during hospitalization (from 98.0% in 2011 to 99.0% in 2021, *p* < 0.001) and the initiation of musculoskeletal rehabilitation within one day after surgery (from 60.4% in 2011 to 81.3% in 2021, *p* < 0.001) increased (Table 2).

### Subgroup analysis

In subgroup analyses based on hospital type, medical costs per hospitalization appeared initially higher in academic hospitals than in non-academic hospitals but seemed to gradually converge, with similar trends observed from 2016 onward (Supplementary Figure S1A). In contrast, daily medical costs per person appeared consistently higher in academic hospitals throughout the study period (Supplementary Figure S1B). Regarding the breakdown of medical costs, expenses for oral drugs, injections, procedures, surgery and anesthesia, laboratory tests, and radiological examinations appeared consistently higher in academic hospitals compared to non-academic hospitals (Supplementary Table S3). Conversely, consultation fees and other resource costs initially higher in academic hospitals in 2011; however, this trend reversed in 2012, with non-academic hospitals subsequently incurring higher hospital fees (Supplementary Table S3).

Subgroup analyses by age showed that medical costs per hospitalization appeared highest in the 70–79 age group, followed by the 80–89, 60–69, and ≥ 90 age groups (Supplementary Figure S2A). Conversely, daily medical costs per person appeared highest in the 60–69 age group, followed by the 70–79, 80–89, and ≥ 90 age groups (Supplementary Figure S2B).

In analyses stratified by sex, both medical costs per hospitalization and daily medical costs per person appeared consistently higher in men than in women (Supplementary Figures S3A and S3B).

## Discussion

In this retrospective cohort study using a nationally representative inpatient database in Japan, we observed that gross medical costs and costs per hospitalization initially declined but gradually increased after 2013 and 2014, respectively. In contrast, daily medical costs per person steadily increased throughout the study period. Notably, both the mean length of hospital stay and waiting times for surgery decreased over time.

Gross medical costs decreased until 2013 but subsequently rose, continuing this upward trend through 2021. This shift closely paralleled trends in both the number of hip fractures and the medical costs per hospitalization, which are key components of gross medical costs. Our findings suggest that the reduction in hip fractures between 2011 and 2013 may have contributed to lower hospitalization and surgical treatment costs during that period. One potential explanation for this decline is the increased use of anti-osteoporotic medications [17, 18]. However, after 2013, the number of hip fractures began to rise, likely driven by Japan’s growing elderly population. In particular, the baby boomer generation surpassed 65 years of age after 2014 [1], increasing the prevalence of osteoporosis and fall-related injuries and, consequently, the incidence of hip fractures and associated medical costs.

Medical costs per hospitalization followed a similar trajectory, decreasing until 2014, likely reflecting gains in acute care efficiency through shorter hospital stays and reduced waiting times. However, this trend reversed after 2014, suggesting that rising costs were driven by factors beyond hospitalization length. A plausible explanation is the increasing complexity of the patient population due to aging and accumulating comorbidities [19]. Our results support this hypothesis, as we observed increases in mean patient age, the proportion of patients with impaired consciousness at admission, and Charlson Comorbidity Index scores over time. These findings indicate that patients with hip fracture have become more medically complex, with greater comorbidity burdens and higher risks of complications. This complexity likely increased demand for diagnostic evaluations, reflected in rising costs for laboratory tests and radiological examinations. Additionally, advances in surgical techniques and medical technologies may have contributed to escalating costs. Specifically, we observed increased use of general anesthesia and total hip arthroplasty, both associated with higher medical expenses [9].

Daily medical costs per person increased steadily throughout the study period. One possible explanation is the progressive shortening of hospital stays, which may have intensified daily resource utilization and thereby increased per-day costs.

Consistent with previous research [3], both the length of hospital stay and waiting time for surgery decreased over the study period. These improvements may be attributed to the regional segmentation of healthcare services and to both international and domestic initiatives, such as the Fragility Fracture Network, Fragility Fracture Network–Japan, and the Toyama Model [4]. However, despite these advancements, Japan’s average hospital stay and surgical waiting times remain substantially longer than those reported in Western countries (Supplementary Figures S4, S5). This persistent disparity likely reflects unique features of the Japanese healthcare system, including the integration of early rehabilitation into acute care settings and systemic barriers to timely transfer from acute hospitals to post-acute care facilities.

Additionally, shifts in discharge patterns may contribute to this disparity, with fewer patients returning home and more being transferred to post-acute care institutions. The proportion of patients discharged directly to their home declined substantially, dropping from 39% in 2011 to 27.5% in 2021. This shift likely reflects the interplay of several contributing factors. First, the increasing age and clinical complexity of hip fracture patients—often accompanied by a greater burden of comorbidities and pre-existing functional impairments—may necessitate continued care beyond the acute hospital setting [20]. Second, the expanding infrastructure for institutional post-acute care in Japan may have facilitated greater transfers to such facilities, particularly when adequate home care support is lacking [4]. Third, rising demand for extended rehabilitation and limited availability of family caregivers may further reduce the feasibility of direct home discharge [21]. These demographic and societal changes underscore the need for more flexible and responsive discharge planning to meet the evolving care needs of hip fracture patients.

Functional recovery at discharge showed a modest decline over the study period, despite the implementation of musculoskeletal rehabilitation in over 98% of patients throughout the study years. Both the proportion of implementation and initiated early rehabilitation (within one day after surgery) demonstrated upward trends. Therefore, this decline in functional recovery is unlikely to be solely attributable to inadequate rehabilitative care. Instead, it may reflect the increasing clinical complexity of the patient population—such as advanced age and multimorbidity—as well as the shortening of hospital stays, which may reduce the time available to achieve sufficient functional gains before discharge, despite early mobilization efforts [22].

Despite substantial postoperative functional decline and frequent discharge to nursing facilities, the in-hospital mortality rate remained below 2%, which appears low. However, this figure may underestimate actual acute to midterm mortality. In Japan, recent efforts have promoted the early transfer of patients from acute hospitals to subacute or long-term care facilities. Deaths occurring after such transfers are not recorded as in-hospital deaths, as the DPC database captures only deaths during the initial hospitalization. Therefore, the reported in-hospital mortality rate may not reflect the true extent of short- to midterm mortality.

Notably, the use of general anesthesia increased steadily throughout the study period. This trend contrasts with international practice, which favors spinal anesthesia in older adults due to its potential benefits, such as reduced postoperative complications and mortality [23, 24]. In Japan, the increased use of general anesthesia may be influenced by systemic factors, including prioritization of operating room efficiency and higher proportion of reimbursement for general anesthesia.

Our analysis also revealed differences in medical costs between academic and non-academic hospitals. Initially, medical costs per hospitalization were higher in academic hospitals but gradually converged with non-academic hospitals after 2016. However, daily medical costs per person remained consistently higher in academic hospitals, potentially due to more significant reductions in hospital stays or a higher proportion of cases requiring intensive resource use. This interpretation is supported by the cost breakdown analysis: while hospitalization fees were lower in academic hospitals, expenses for drugs, diagnostics, and procedures were consistently higher. This pattern suggests that academic hospitals treat more complex cases requiring additional tests, treatments, and procedures, thereby consuming more resources during acute care.

The reduction in hospital stay duration and surgical waiting times observed during the study period suggests improved efficiency in the acute management of hip fractures. However, the concurrent rise in medical costs associated with hip fracture treatment is likely attributable to demographic shifts, particularly the increasing number of older adults requiring care. As many countries are experiencing similar trends in population aging, the Japanese experience may offer valuable insights. Therefore, future health policies should consider both the effectiveness of disease-specific treatment and the long-term sustainability of healthcare expenditures in aging societies.

This study has several limitations. First, while annual gross medical costs and the number of hip fractures were adjusted for the number of patients registered in the DPC system, demographic changes over time were not directly accounted for in the trend analyses. Second, because participation in the DPC database was voluntary and non-DPC hospitals were excluded, the generalizability of our findings may be limited. Third, we could not adjust for institutional characteristics, as the database lacked relevant information. The institution-specific medical facility coefficient, which changes annually, may have influenced trends in hospitalization fees. Fourth, our analysis did not capture long-term medical costs, such as outpatient care and rehabilitation, or indirect costs like productivity loss, potentially underestimating the total economic burden of hip fractures. Fifth, the study excluded patients with hip fractures who did not undergo surgery. Finally, trends related to delirium screening were not assessed, as the DPC database lacks detailed information on this measure. Delirium is a common and serious complication following hip fracture, known to affect outcomes such as length of hospital stay, mortality, and discharge disposition [25]. As such, the trends and outcomes reported in this study may have been implicitly influenced by unmeasured delirium incidence and its management.

In conclusion, this study demonstrated that, despite improvements in hospital efficiency, acute care costs for hip fractures showed varying trends. Specifically, gross medical costs and costs per hospitalization initially declined but began increasing after 2013 and 2014, respectively. In contrast, daily medical costs per person consistently rose throughout the study period. These findings underscore the growing economic burden of hip fracture care and highlight the need for strategic policy interventions to achieve sustainable healthcare delivery.

## Supplementary Information

Below is the link to the electronic supplementary material.

Supplementary file 1 (DOCX 149 KB)

## Data Availability

Authors confirm the availability of data supporting the findings of this study within the article and supplementary materials.
