# Supplementary material for: Temporal trends in acute care costs of hip fracture treatment from 2011 to 2021 in Japan
Source: Arch Osteoporos. 2025 Sep 12;20(1):124. doi: 10.1007/s11657-025-01607-3 (PMC12432063; doi:10.1007/s11657-025-01607-3)
Supplement: Supplementary file 1 — (DOCX 149 KB) [file 11657_2025_1607_MOESM1_ESM.docx]

**Supplementary Online Content**

**Table S1. List of Japanese original procedure codes for inclusion criteria**

**Table S2. List of ICD-10 codes for exclusion criteria**

**Table S3. Trends in medical cost breakdown per hospitalization, stratified by institutional level, 2011–2021**

**Figure S1. Annual changes in medical costs, stratified by institutional level, 2011–2021**

**Figure S2. Annual changes in medical costs, stratified by age, 2011–2021**

**Figure S3. Annual changes in medical costs, stratified by sex, 2011–2021**

**Figure S4. International overview of the mean length of hospital stay, 2019**

**Figure S5. International overview of the mean waiting times for surgery, 2019**

**Table S1. List of Japanese original procedure codes for inclusion criteria**

| **Inclusion criteria** | **Original Japanese procedure codes** |
| --- | --- |
| Total hip arthroplasty | K0821 |
| Hemiarthroplasty | K0811 |
| Open reduction internal fixation | K0461, K0731 |

**Table S2. List of ICD-10 codes for exclusion criteria**

| **Exclusion criteria** | **ICD-10 codes of main diagnoses, admission precipitating diagnoses, or comorbidities present on admission** |
| --- | --- |
| Multiple traumas | T07 |
| Open fracture | T1421, S7201, S7211, S7221, S7231, S7271, S7291 |
| Pathological fracture | M8445, M8449 |

ICD-10, International Classification of Diseases, 10th Revision

**Table S3. Trends in medical cost breakdown per hospitalization, stratified by institutional level, 2011–2021**

|  | **2011** | **2012** | **2013** | **2014** | **2015** | **2016** | **2017** | **2018** | **2019** | **2020** | **2021** | **p-value** |
| --- | --- | --- | --- | --- | --- | --- | --- | --- | --- | --- | --- | --- |
| **Academic hospitals** |  |  |  |  |  |  |  |  |  |  |  |  |
| Consultation fee | 94 (114) | 91 (119) | 89 (85) | 93 (107) | 94 (84) | 89 (80) | 93 (105) | 89 (68) | 88 (71) | 91 (69) | 98 (87) | <0.001 |
| Oral drugs | 142 (268) | 126 (279) | 120 (192) | 126 (316) | 117 (256) | 126 (281) | 123 (287) | 140 (1003) | 121 (452) | 117 (387) | 125 (412) | <0.001 |
| Injection | 365 (1000) | 368 (1712) | 384 (1360) | 363 (2324) | 323 (1210) | 270 (862) | 216 (579) | 257 (1392) | 202 (623) | 199 (525) | 196 (560) | <0.001 |
| Procedure | 273 (1069) | 228 (836) | 246 (1228) | 223 (881) | 215 (815) | 192 (706) | 188 (696) | 172 (648) | 185 (662) | 184 (678) | 177 (648) | <0.001 |
| Surgery and anesthesia | 5284 (2665) | 5446 (2772) | 5443 (2785) | 5128 (2711) | 5115 (2702) | 4957 (2362) | 4956 (2689) | 5032 (2844) | 5045 (2752) | 4934 (2684) | 5050 (2933) | <0.001 |
| Laboratory tests | 578 (316) | 549 (356) | 548 (396) | 527 (315) | 519 (375) | 512 (301) | 509 (279) | 512 (298) | 499 (294) | 562 (320) | 611 (310) | <0.001 |
| Radiological examination | 316 (260) | 322 (276) | 324 (246) | 321 (228) | 328 (257) | 332 (232) | 317 (202) | 317 (212) | 313 (209) | 328 (200) | 331 (204) | <0.001 |
| Other resources | 368 (293) | 354 (378) | 370 (420) | 359 (411) | 352 (382) | 375 (365) | 372 (425) | 500 (4174) | 381 (588) | 397 (713) | 392 (590) | <0.001 |
| Hospital fee | 5191 (2575) | 4915 (2877) | 4780 (2750) | 4708 (2575) | 4595 (2853) | 4552 (2397) | 4408 (2207) | 4382 (2529) | 4438 (2474) | 4455 (2384) | 4676 (2533) | <0.001 |
| **Non-academic hospitals** |  |  |  |  |  |  |  |  |  |  |  |  |
| Consultation fee | 143 (111) | 139 (112) | 139 (109) | 135 (109) | 137 (107) | 132 (114) | 131 (106) | 134 (107) | 134 (110) | 139 (106) | 144 (112) | 0.985 |
| Oral drugs | 61 (122) | 63 (117) | 64 (205) | 59 (121) | 58 (161) | 54 (138) | 53 (123) | 50 (127) | 50 (122) | 50 (172) | 49 (129) | <0.001 |
| Injection | 193 (558) | 181 (571) | 179 (627) | 162 (520) | 159 (454) | 152 (1056) | 137 (483) | 121 (398) | 120 (373) | 128 (1358) | 126 (832) | <0.001 |
| Procedure | 134 (641) | 140 (810) | 139 (2313) | 122 (579) | 121 (625) | 118 (583) | 115 (796) | 114 (631) | 110 (560) | 110 (677) | 111 (643) | <0.001 |
| Surgery and anesthesia | 4653 (2232) | 4779 (2145) | 4725 (2180) | 4574 (2028) | 4445 (1949) | 4339 (1830) | 4347 (1895) | 4299 (1795) | 4328 (1849) | 4413 (1901) | 4461 (1907) | <0.001 |
| Laboratory tests | 310 (226) | 321 (236) | 321 (233) | 317 (211) | 324 (222) | 329 (218) | 333 (218) | 333 (213) | 334 (214) | 380 (237) | 436 (249) | <0.001 |
| Radiological examination | 212 (167) | 223 (168) | 228 (168) | 228 (276) | 236 (161) | 242 (155) | 245 (150) | 247 (146) | 250 (140) | 266 (146) | 267 (143) | <0.001 |
| Other resources | 723 (1002) | 760 (1089) | 757 (1182) | 762 (1110) | 780 (1150) | 830 (1240) | 835 (1269) | 870 (1342) | 851 (1297) | 892 (1314) | 878 (1293) | <0.001 |
| Hospital fee | 5156 (3161) | 5167 (3142) | 5062 (3175) | 4977 (2979) | 5101 (3359) | 5243 (3403) | 5289 (3609) | 5361 (3436) | 5427 (3508) | 5500 (3667) | 5567 (3617) | <0.001 |


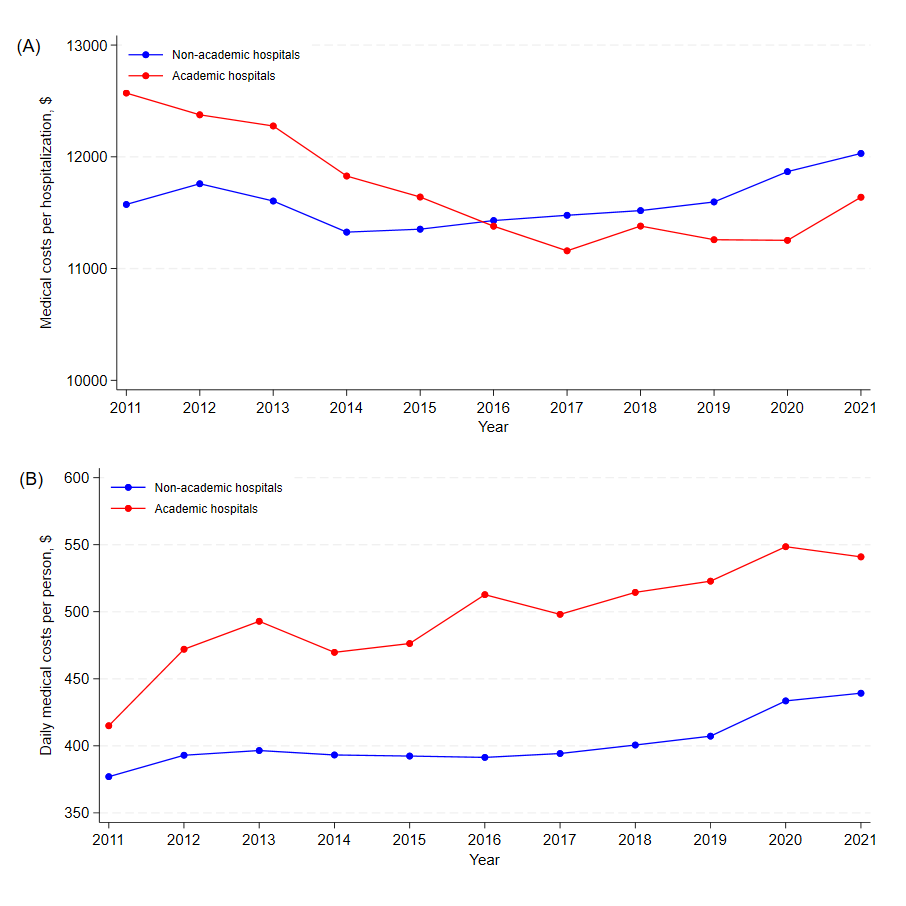


**Figure S1. Annual changes in medical costs, stratified by institutional level, 2011–2021**

**(A) Annual changes in medical costs per hospitalization**

**(B) Annual changes in daily medical costs per person**

**
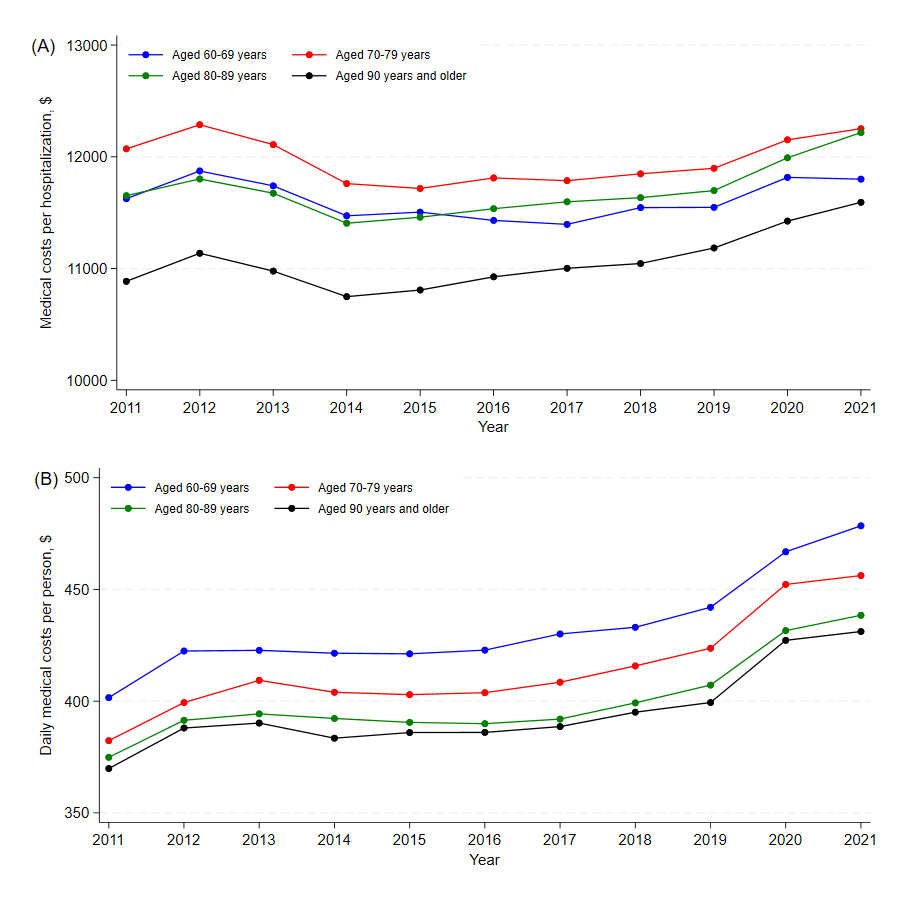
**

**Figure S2. Annual changes in medical costs, stratified by age, 2011–2021**

**(A) Annual changes in medical costs per hospitalization**

**(B) Annual changes in daily medical costs per person**

**
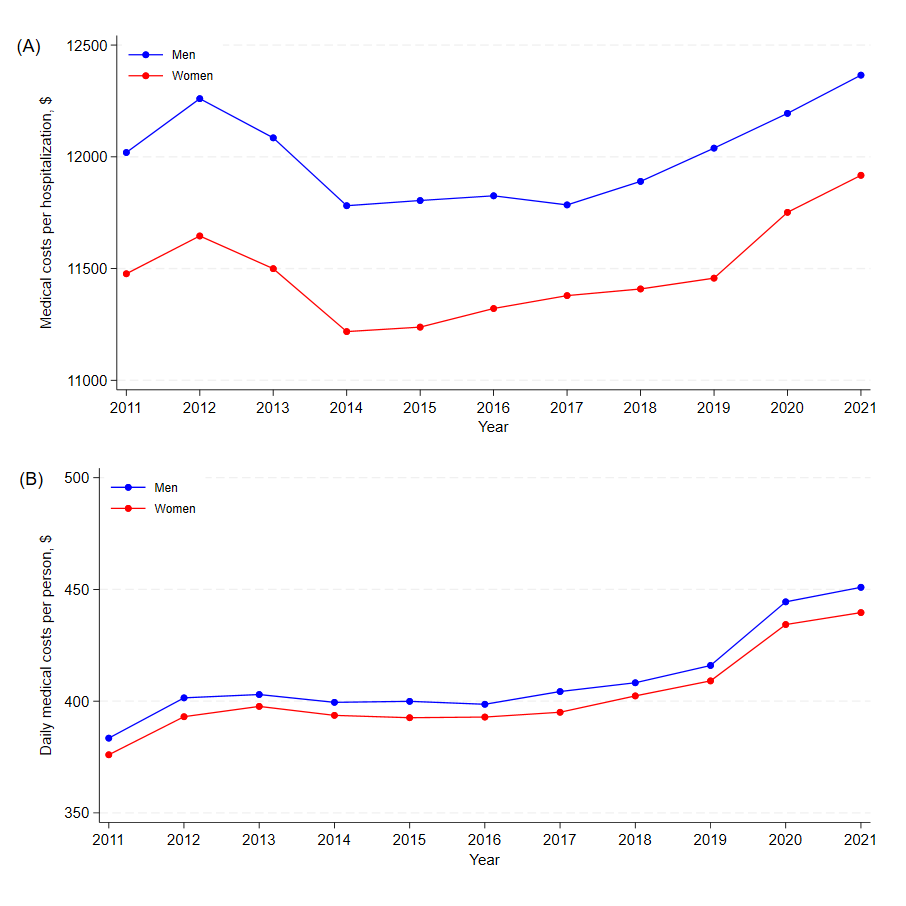
**

**Figure S3. Annual changes in medical costs, stratified by sex, 2011–2021**

**(A) Annual changes in medical costs per hospitalization**

**(B) Annual changes in daily medical costs per person**

**Figure S4. International overview of the mean length of hospital stay, 2019**

**Figure S5. International overview of the mean waiting times for surgery, 2019**
